# Mobile Right Atrial Thrombi in a Patient with the Hemoglobin SC Disease

**DOI:** 10.1155/2011/897167

**Published:** 2011-09-06

**Authors:** H. O. Savage, N. Ding, O. Eso, B. Sachdev, D. L. Lefroy

**Affiliations:** ^1^Department of Cardiology, Hammersmith Hospital, Imperial College Healthcare NHS Trust, Du Cane Road, London W12 0NN, UK; ^2^Department of Medicine, Colchester University Hospital Foundation Trust, Turner Road, Colchester, Essex CO4 5JL, UK; ^3^Department of Medicine, University of Bristol Medical School, Avon BS8 1UQ, UK

## Abstract

The formation of Intracardiac thrombi is rare in the absence of structural heart disease or atrial fibrillation. We describe a case of spontaneous right atrial thrombus formation that occurred in a patient with a hypercoagulable condition who had been sub optimally anticoagulated.

## 1. Introduction

Sickle cell disease (SCD) is an inherited autosomal recessive disorder characterized by the formation of haemoglobin S (HbS) resulting from the amino acid substitution of glutamate to valine on the beta chain of haemoglobin. The vasoocclusive and haemolytic complications of this disease are well known however it is worth noting that there are clinical manifestations of this hypercoagulable disease such as large vessel thrombosis, and in rare cases, intracardiac thrombus.

We report a case of spontaneous right atrial thrombus formation in a patient with sickle cell disease who had been suboptimally anticoagulated.

## 2. Case Report

A 52-year-old man with haemoglobin SC disease, a previous surgical splenectomy 26 years prior, following a crisis, and pulmonary arterial hypertension secondary to chronic thromboembolic disease, presented with acute dyspnoea and pleuritic chest pain. Four days earlier, he had run out of warfarin and had neglected to renew his prescription. His admission ECG showed anterolateral ST depression and his troponin I measured 16 *μ*g/L. The international normalized ratio was 1.1. He was hypotensive with a systolic blood pressure of 95 mmHg and tachycardic with a pulse rate of 120 per minute with low oxygen saturations at 88%. His hemoglobin concentration was measured at 9.0 g/dL. 

An urgent computed tomographic pulmonary angiography did not demonstrate fresh pulmonary emboli. Exchange blood transfusion was undertaken because of low oxygen saturations the day after admission but this did not improve the situation. He continued to complain of chest pains and subsequently had an episode of collapse two days later with further T wave and ST ECG changes. 

**Figure 1:**
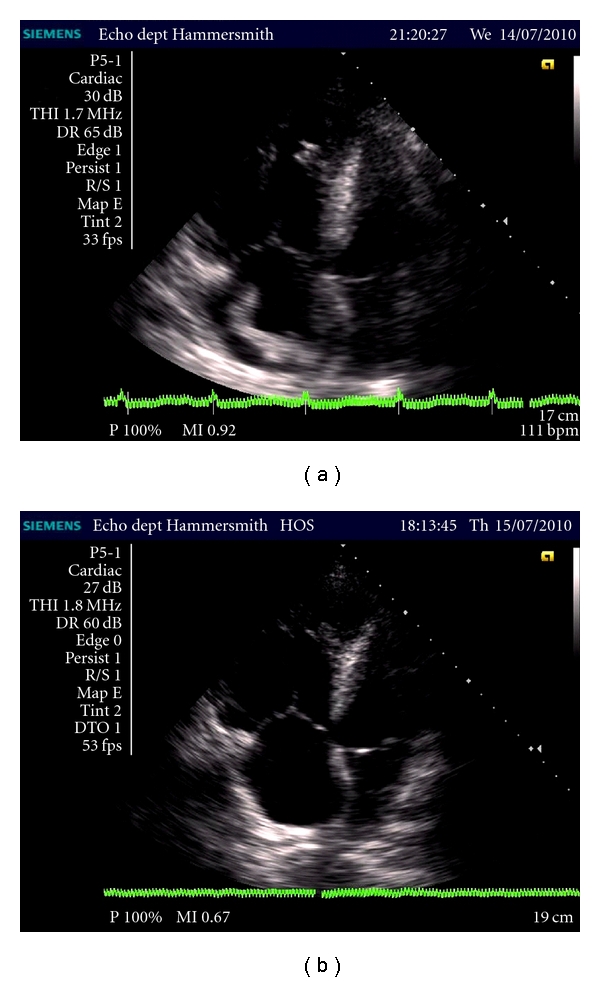
(a) shows “snake-like” thrombus within right atrium. (b) shows dissolution of thrombus post fibrinolytic agent.

Transthoracic echocardiography (TTE) demonstrated a large thrombus within the right atrium (Figure 1(a)). This was moving freely into the right ventricle, the pulmonary artery, and the inferior vena cava. There was no clinical evidence of deep vein thrombosis.

The patient received thrombolysis with an initial bolus then infusion of alteplase. Repeat TTE 21 hours later demonstrated disappearance of the thrombus (Figure 1(b)). The patient was restarted on warfarin and recovered. 

## 3. Discussion

Spontaneous right atrial thrombus formation is rare in patients without structural right heart disease or atrial fibrillation. Mobile right-sided thrombi, in particular, are thought to be a severe form of venous thromboembolism, arising from deep vein thrombosis [1]. These can produce emboli at any time and are associated with a high mortality rate greater than 40% [2]. Immediate treatment is therefore imperative.

Right heart thrombi have been identified in diseases of hypercoagulability such as protein C and S deficiency [3], Behcets disease [4, 5], and inflammatory bowel disease [6]. It remains a very rare presentation of sickle cell disease but we have found one reported case in literature [7]. In an autopsy case series of fourteen patients with sickle cell disease that died within a 15-year period, right atrial thrombosis was found in one patient who had presented ante mortem with an acute chest syndrome [8]. 

SCD predisposes to hypercoagulability due to abnormal activation of the fibrinolytic system by abnormal sickle cells which activate the prothrombinase complex resulting in production of high plasma levels of thrombin. Depletion of anticoagulation factors, increased tissue factor expression, and chronic platelet activation also play an important role [9]. 

Splenectomy in haematological diseases such as Beta thalassaemia intermedia and SCD, have been thought to further contribute to this observed hypercoagulable state and subsequent thromboembolic complications, due to the procoagulant activity of a high number of circulating damaged RBC, thrombocytosis, and activation of the coagulation system which occur [10, 11]. Unlike the Beta thalassaemias however, there is currently little in terms of literature which shows this direct association between sickle cell disease and splenectomy in particular [12]. 

Up to 30% of patients with SCD will develop pulmonary arterial hypertension (PAH) and this is known to be associated with significant morbidity and mortality. Several mechanisms are recognised in the pathogenesis of PAH which include recurrent hemolysis, nitric oxide deficiency, vascular bed remodeling, and chronic hypoxaemia [13, 14]. Recurrent thromboembolic disease is also thought to contribute and undoubtedly some of such cases can be attributed to occult thromboembolism caused by intracardiac thrombi.

Though SCD is a hypercoaguable state, the roles of anticoagulants have not been well defined and larger controlled trials are required to define their role [9]. As mentioned, chronic pulmonary thromboembolism is a recognized association and in such cases it is generally acceptable to administer oral anticoagulant therapy and there is the suggestion that low dose anticoagulation in these patients may reverse some of the prothrombotic phenomena [15]. Our patient was already on warfarin due to chronic small vessel pulmonary thromboembolism but was Suboptimal from noncompliance issues. Suboptimal anti-coagulation and the risk of intracardiac thrombus formation have already been described in other cases of high-risk patients for intracardiac thrombi formation, such as cardiac surgery [16].

The importance of transthoracic echocardiogram as a key simple, noninvasive, and rapid investigation for diagnosing intracardiac thrombi is highlighted here. A retrospective study by Chartier et al. of 38 patients with mobile right-heart thrombi also underlines the usefulness of TTE as a first-line investigation [1] allowing rapid life-saving treatment to be initiated. Felner et al. in a small study of patients with clinically unsuspected right atrial thrombemboli, found that TTE was instrumental in making a diagnosis and providing information on the size, shape, mobility, and location of subsequently demonstrated intracardiac thrombi in these patients [17]. In more recent studies transoesophageal echocardiogram has been noted to be particularly invaluable in the diagnosis of right-sided cardiac masses and where available may be a preferred option with a high diagnostic yield achievable [18, 19].

There is no clear consensus of the preferred treatment option and until a randomized control trial is embarked on, the debate will continue. Factors that have been considered however include extent, size, shape, and mobility of the cardiac thrombus, preexisting pulmonary embolism/deep vein thrombosis, and cardiopulmonary reserve [3, 20]. Chartier's study did not delineate a statistical difference between various treatment modalities and mortality; these included surgical thrombectomy, intravenous thrombolysis, intravenous heparin, and percutaneous intervention. However it is worth noting that in the European Cooperative Study, the heparin treated group registered a high mortality rate [1, 21]. In general, patients with intracardiac thrombi who present with coexisting pulmonary embolism and/or hemodynamic embarrassment seem to do better with thrombolytic therapy. This represents a safe and effective option with rapid resolution of thrombus, improvement of hemodynamic status, and echocardiographic parameters of acute right ventricular overload [22]. Complication rates were also noted to be infrequent [23].

Mortality in patients with intracardiac thrombi remains high. Chartier et al. study found that a fifth of their patients diagnosed with right atrial thrombi died within 24 hours off admission [1]. In a review by the European working group of echocardiography, they observed that patients with Type A thrombi (long, thin, extremely mobile thrombi which resembled a worm or a snake), which is similar to what our patient presented with, had early mortality (< or equal to 8 days) in excess of 42% and overall prognosis was generally poor. The presence of pulmonary embolism on its own in association with right heart thromboembolism was found to be a poor prognostic indicator. This further emphasizes the need for rapid diagnosis and emergency treatment to prevent mortality [2].

## 4. Conclusion

Sickle cell disease as a hypercoaguable state is a rare risk factor for intracardiac thrombus. A high index of suspicion is therefore needed to make a diagnosis. TTE can provide a rapid diagnosis of intracardiac thrombosis and influence immediate life saving management. In this instance, intravenous thrombolytic therapy offered a safer and more acceptable alternative to surgical removal of the thrombus.
